# Supplementary material for: The overlapping global distribution of dengue, chikungunya, Zika and yellow fever
Source: Nat Commun. 2025 Apr 10;16:3418. doi: 10.1038/s41467-025-58609-5 (PMC11986131; doi:10.1038/s41467-025-58609-5)
Supplement: Supplementary file 1 — Supplementary Information [file 41467_2025_58609_MOESM1_ESM.pdf]

Supplementary Information for:

The overlapping global distribution of dengue, chikungunya, Zika and yellow fever

Contents

Supplementary Table 1. Data sources and the number of occurrence data extracted from each source before the removal of spatial duplicates (thinning). 2

Supplementary Table 2. Number of occurrence points for each arboviral disease before and after the removal of spatial duplicates (thinning). 3

Supplementary Table 3. Number of occurrence points for acute viral infectious diseases before and after the removal of spatial duplicates (thinning). 4

Supplementary Table 4. Covariates included in the models. 5

Supplementary Table 5. Questionnaire for independent validation of preliminary results presented to the Technical Advisory Group on Arbovirus (TAG-Arbovirus) 6

Supplementary Table 6. Comparison of model performance metrics for individual disease models vs. joint disease model 7

Supplementary Figure 1. Comparing occurrence data for each disease between published and newly added datasets for each disease. 8

Supplementary Figure 2. Occurrence point data for all viral diseases which are used in the surveillance model (post thinning). 9

Supplementary Figure 3. Contribution of each covariate to the global surveillance capability model. 10

Supplementary Figure 4. Monotonic spline fits showing the partial effects of individual covariates on surveillance capability with 95% confidence intervals. 11

Supplementary Figure 5. The degree of uncertainty around surveillance capability model predictions. 12

Supplementary Figure 6. Overall and regionally-stratified model performance metrics for the global surveillance capability model 13

Supplementary Figure 7. Spatial map illustrating the model performance of the global surveillance capability model. 14

Supplementary Figure 8. Contribution of each covariate to the arbovirus model (dengue, chikungunya, and Zika). 15

Supplementary Figure 9. Model predicted environmental suitability of each of dengue, chikungunya, and Zika. 16

Supplementary Figure 10. A comparison of arbovirus model performance with and without disease-specific thermal suitability included as a covariate. 17

Supplementary Figure 11. The degree of uncertainty around arbovirus model predictions. 18

Supplementary Figure 12. Overall and regionally-stratified model performance metrics for the arbovirus and yellow fever models 19

Supplementary Figure 13. Spatial map illustrating the model performance of the arbovirus model. 20

Supplementary Figure 14. Contribution of each covariate to the yellow fever model. 21

Supplementary Figure 15. A comparison of yellow fever model performance with and without *Aedes aegypti* included as a covariate. 22

Supplementary Figure 16. Covariates included in the surveillance capability model. 23

Supplementary Figure 17. Covariates included in the arbovirus and yellow fever models. 24

Supplementary Figure 18. Masking layers for arbovirus and yellow fever prediction. 25

Supplementary Figure 19. Model predicted environmental suitability of arboviruses without masking. 26

Supplementary Figure 20. Comparisons of maps using occurrence points pre- (up to 2014) and post-Zika epidemic (up to 2024) 27

References 28

Supplementary Table 1. Data sources and the number of occurrence data extracted from each source before the removal of spatial duplicates (thinning).

Disease	Data	Number of occurrence data	Years covered	Source
Acute viral diseases	Published dataset	69,426	2006-2014	[1]
	HealthMap	385,944	2006-2019	www.healthmap.org
Dengue	Published dataset	13,604	1960-2015	[2]
	HealthMap	23,527	2015-2019	www.healthmap.org
	Others	96	2015-2024	[3–8]
Chikungunya	Published dataset	1,211	1952-2015	[9]
	HealthMap	1,330	2015-2019	www.healthmap.org
	ProMed mail	531	2015-2022	
	Others	10,974	2015-2024	[10–13]
Zika	Published dataset	237	1953-2016	[14]
	HealthMap	3,398	2015-2019	www.healthmap.org
	ProMed mail	773	2015-2022	
Yellow fever	Published dataset	1,175	1927-2016	[1]
	HealthMap	1,400	2015-2019	www.healthmap.org
	Others	105	2015-2024	[4,15]

Supplementary Table 2. Number of occurrence points for each arboviral disease before and after the removal of spatial duplicates (thinning).

	Dengue	Chikungunya	Zika	Yellow fever
Before thinning	37227	14046	4408	2680
After thinning	5867	4727	1138	1395

Supplementary Table 3. Number of occurrence points for acute viral infectious diseases before and after the removal of spatial duplicates (thinning).

Disease	Before thinning	After thinning
Avian influenza H5N1	5036	583
Chicken pox	4069	310
Chikungunya	15113	668
Common cold	1452	93
Crimean-Congo haemorrhagic fever	940	166
Dengue	130907	7222
Eastern equine encephalitis	2819	307
Enterovirus infection	972	59
Hepatitis A	4649	293
Hepatitis B	2008	88
Hepatitis C	4157	125
Hepatitis D	70	0
Hepatitis E	543	42
Herpes	703	42
Influenza	81755	6052
Japanese encephalitis	1991	105
La Crosse encephalitis	148	26
Lassa fever	2319	107
Machupo virus	12	3
Marburg fever	206	17
Mononucleosis	162	19
MPOX	383	41
Mumps	5573	239
Murray Valley encephalitis	106	9
Nipah and Nipah-like virus disease	893	49
Parvovirus	1135	159
Powassan	288	10
Rabies	22040	2081
Respiratory syncytial virus	789	21
Rift Valley fever	988	99
Roseola	9	1
Ross River virus	326	30
Rotavirus	1008	78
Rubella	1746	84
Shingles	191	13
St. Louis encephalitis	242	8
Tick-borne encephalitis	416	47
Venezuelan equine encephalitis	169	10
Viral meningitis	863	155
West Nile fever	26420	1737
Western equine encephalitis	2	1
Yellow fever	4247	278
Zika	10140	223

Supplementary Table 4. Covariates included in the models.

	Covariate	Terms in Equations (1) and (2)	Years covered	Original spatial resolution	Data source
Surveillance factors	Gross Domestic Product (5km x 5km and aggregated national level)	GDP and $GDP_{National}$	2009-2019	1 km	[16]
	Proportion of urban land	$Urban$	2010 and 2020	1 km	[17]
	Travel time to healthcare facilities (walking)	$travel_{health}$	2020	1 km	[18]
	Travel time to cities (>50,000 people, any travel mode)	$travel_{cities}$	2015	1 km	[18]
	Proportion of febrile children who sought treatment at any (public or private) healthcare facility	$treatmentseeking$	2010-2022	National	[19]
	Child mortality under five years old	$childmortality$	2013-2020	National	[20,21]
	Government effectiveness	$goveffectiveness$	2013-2020	National	[21,22]
	Physicians density	$physician$	2010-2018	National	[21,23]
Transmission factors	Temperature suitability for dengue virus transmission	$Temp$	2010-2020	5 km	[24]
	Mean temperature of the coldest month	$Tcold$	2010-2020	5 km	[25]
	Average of annual precipitation	$Precip$	2010-2020	5 km	[25]
	Normalized Difference Vegetation Index (NDVI)	$NDVI$	2010-2020	5 km	[26]
	Dynamic Habitat Indices (DHI)	DHI	-	1 km	[27]
	Predicted suitability for *Aedes aegypti*	$aegypti$	2020	5 km	[28]
	Predicted suitability for *Aedes albopictus**	$albo$	2020	5 km	[28]
	Gross Domestic Product (aggregated national level)	$GDP_{National}$	2009-2019	National	[16]
	Human population density	Pop	2022	5 km	[29]
	Predicted suitability for *Haemagogus janthinomys***	$Haemagogus$	2020	1 km	[30]
	Non-human primate distribution**	NHP	2017	5 km	[1]
	Yellow fever vaccination coverage**	$Vaccine$	2020	Admin1	[31]

* included in combined dengue, chikungunya, and Zika model only. ** included in yellow fever model only.

Supplementary Table 5. Questionnaire for independent validation of preliminary results presented to the Technical Advisory Group on Arbovirus (TAG-Arbovirus)

Category	#	Question
Data	1	Are you aware of any additional locations where any of these arboviral diseases are present?
Surveillance capability map	2	Do our estimates of relative surveillance intensity for viral pathogens within and between countries match your expectations?
	3	Are there any additional drivers of surveillance capability?
Arbovirus maps	4	Any areas where any of these diseases are present, but where we predict absence?
	5	Any areas where any of these diseases are absent, but where we predict presence?
	6	Additional drivers of arbovirus risk?
	7	Are there any differences in the distribution of these diseases that we have not captured in these risk maps?

Supplementary Table 6. Comparison of model performance metrics for individual disease models vs. joint disease model

	AUC		Sensitivity		Specificity	
	Individual	Joint	Individual	Joint	Individual	Joint
Dengue	0.982	0.984	0.936	0.940	0.935	0.935
Chikungunya	0.992	0.994	0.970	0.974	0.955	0.957
Zika	0.992	0.995	0.969	0.978	0.945	0.961
Yellow fever	0.977	0.977	0.962	0.929	0.912	0.922

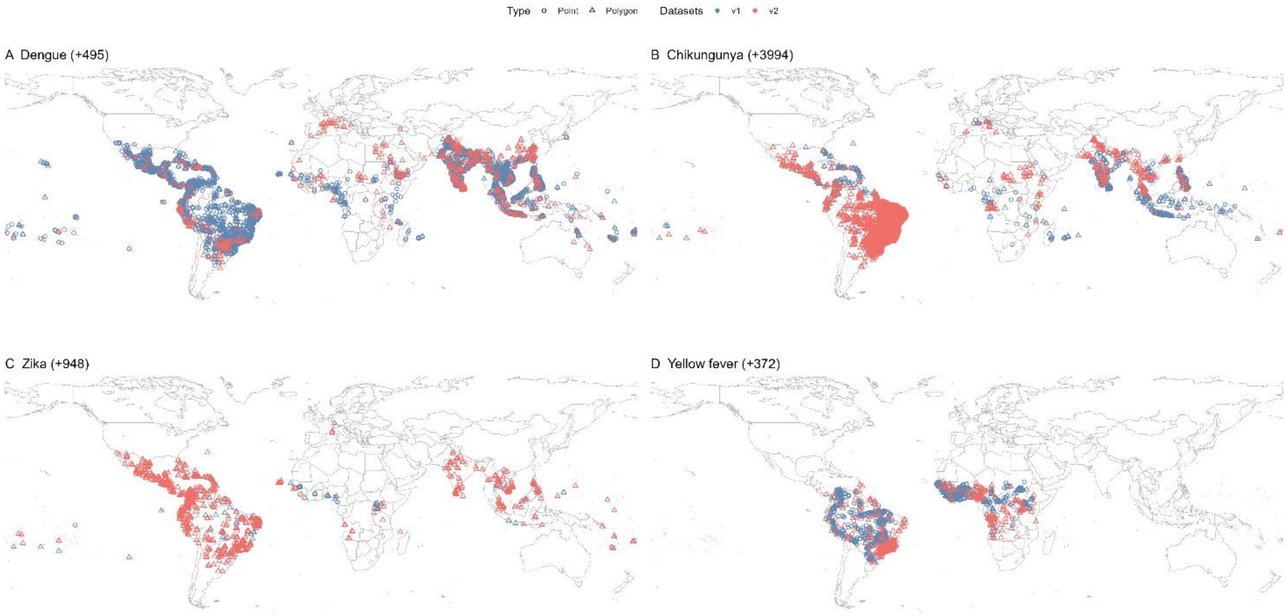

Supplementary Figure 1. Comparing occurrence data for each disease between published and newly added datasets for each disease.

Maps show the unique locations of occurrence data for dengue (A), chikungunya (B), Zika (C), and yellow fever (D). These data combine existing datasets (blue) with new additions identified through this study (red). The number of newly added unique occurrence locations for each disease is indicated in parentheses. The maps were created using public-domain Natural Earth data, accessed through the rnaturalearth package in R[32].

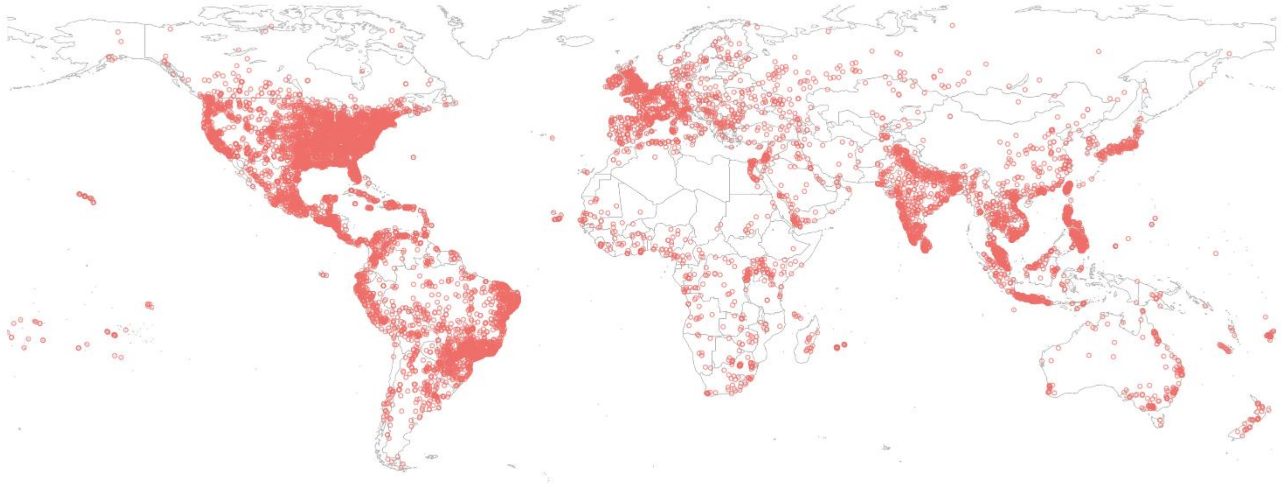

Supplementary Figure 2. Occurrence point data for all viral diseases which are used in the surveillance model (post thinning).

The dataset includes occurrence records from HealthMap (2006–2019), excluding diseases with dedicated surveillance programs (Ebola, HIV/AIDS, measles, and polio) to avoid bias. A total of 338,005 records were filtered and standardised, resulting in 21,700 records after thinning. The final dataset focuses on viral diseases causing acute febrile illness, primarily diagnosed by serology and PCR. See Supplementary Table 3 for disease counts before and after thinning. The map was created using public-domain Natural Earth data, accessed through the rnaturalearth package in R[32].

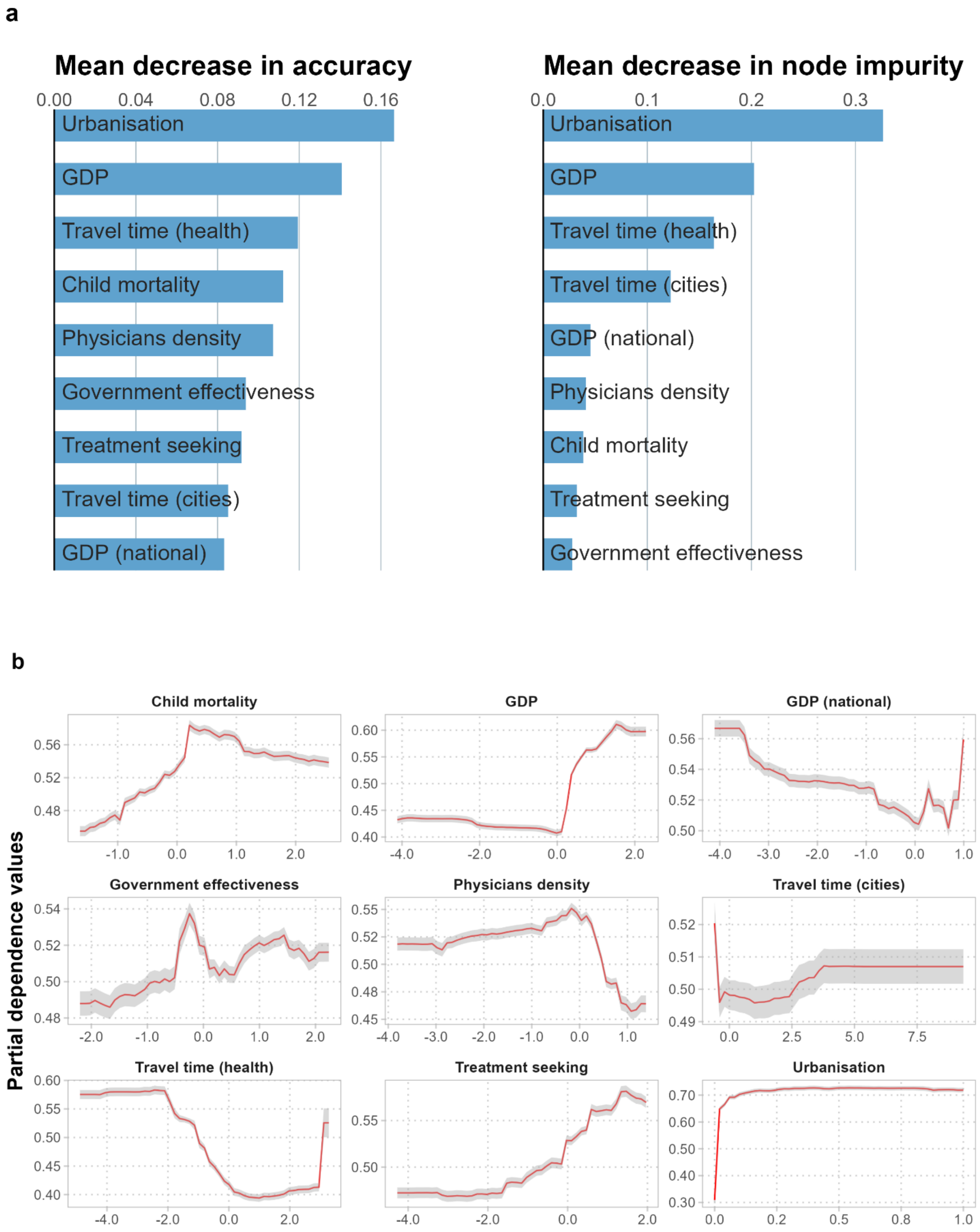

Supplementary Figure 3. Contribution of each covariate to the global surveillance capability model.
Relative contribution of each covariate in explaining the global surveillance capability (a); Partial dependence plots showing the effects of each covariate on the overall response with 95% confidence intervals, where higher y-axis values indicate greater surveillance capability (b).

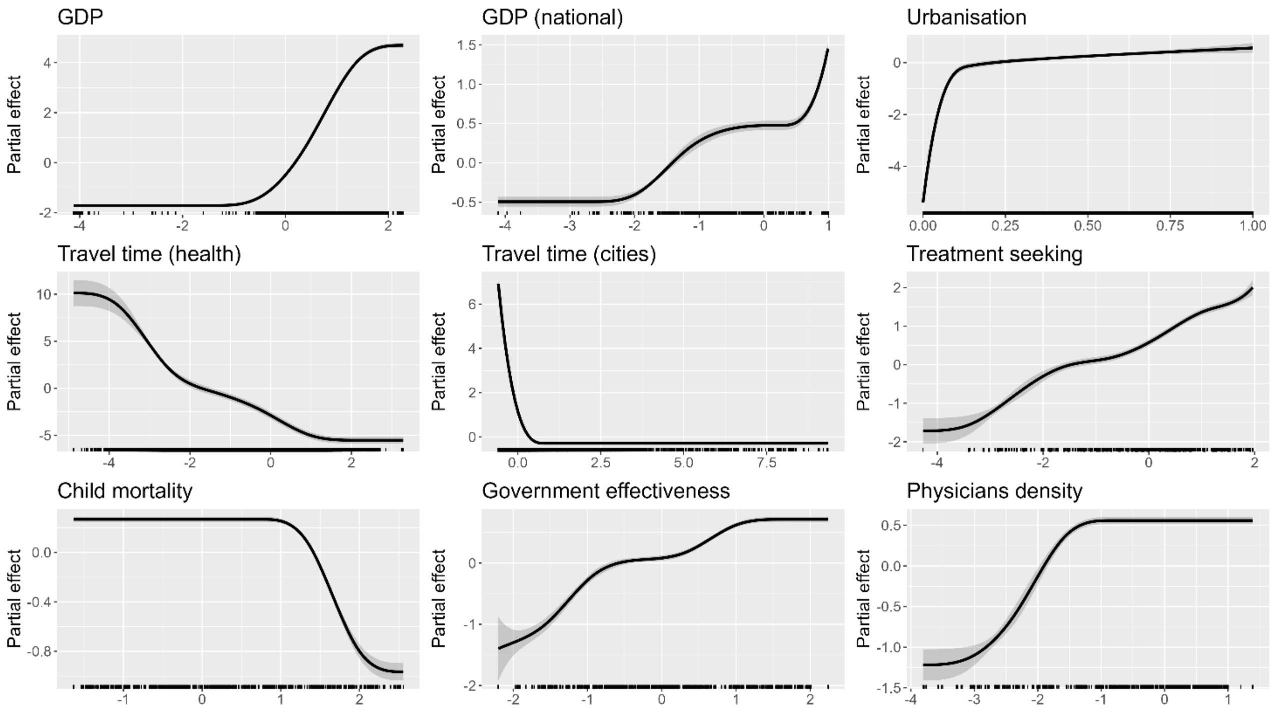

Supplementary Figure 4. Monotonic spline fits showing the partial effects of individual covariates on surveillance capability with 95% confidence intervals.

Generalised additive models (GAMs) were fitted for each covariate using spline fits with monotonic constraints, where higher y-axis values indicate greater surveillance capability. Partial effect plots show that higher physician density and GDP are associated with increased surveillance, while lower child mortality also corresponds to higher surveillance.

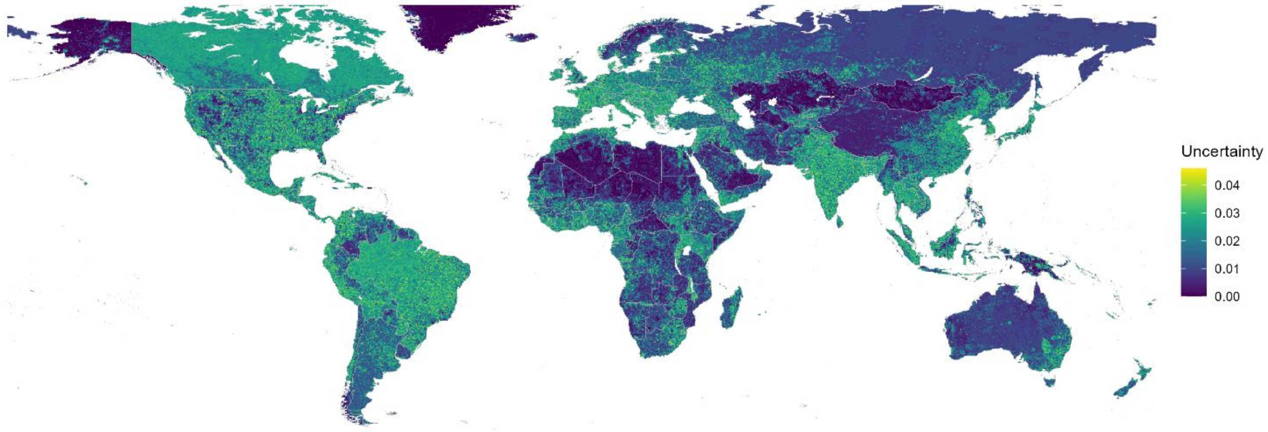

Supplementary Figure 5. The degree of uncertainty around surveillance capability model predictions.

A total of 100 random forest sub-models were calibrated through spatial cross-validation to predict surveillance capability scores (0-1 scale). Model uncertainty was quantified by calculating the interquartile range (IQR) of the 100 model predictions at each location. The map was created using public-domain Natural Earth data, accessed through the rnaturalearth package in R[32].

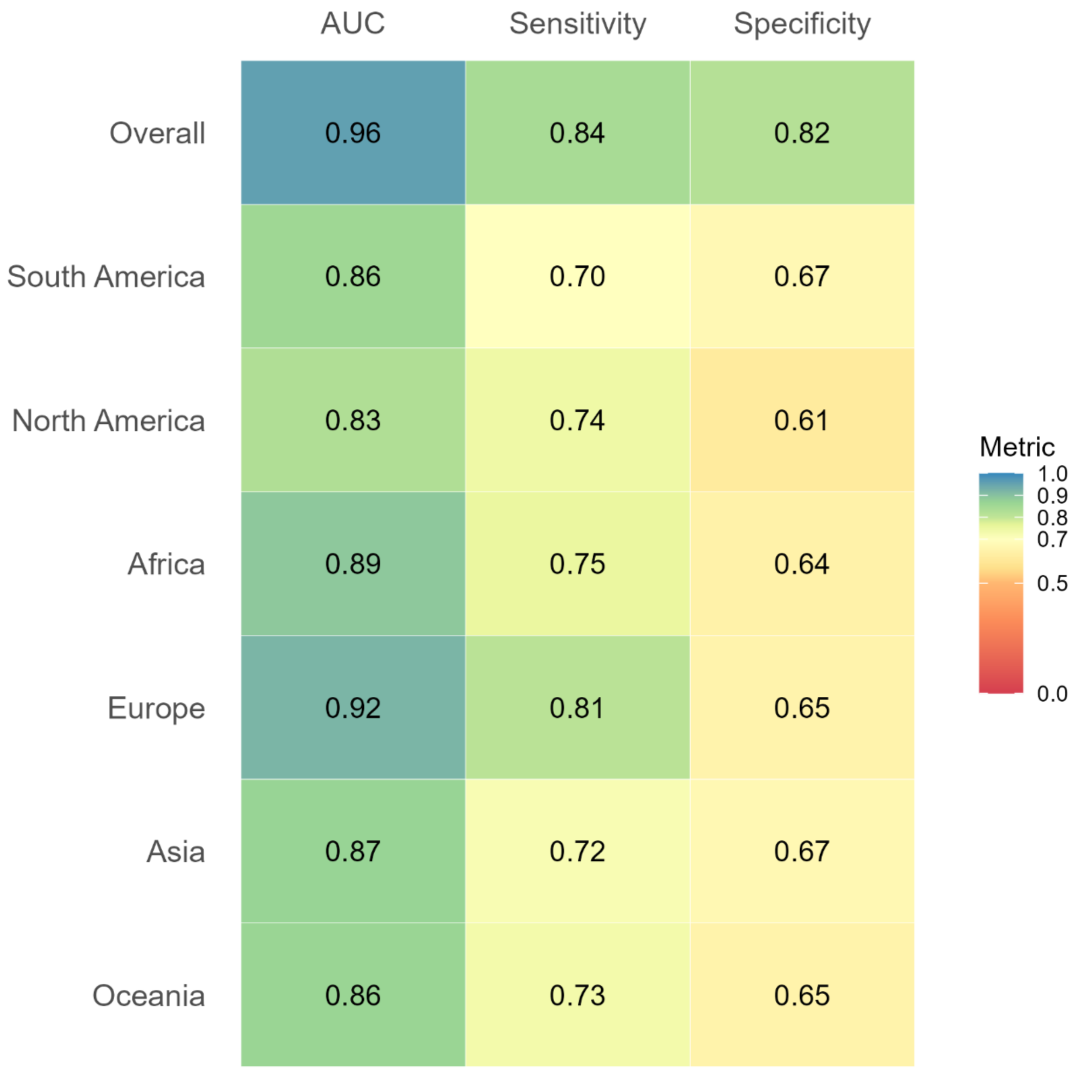

Supplementary Figure 6. Overall and regionally-stratified model performance metrics for the global surveillance capability model

Area Under the Curve (AUC), sensitivity, and specificity were calculated for each validation polygon (250 km radius around each presence or background point). Global and regionally stratified values for each metric were then derived by averaging these values within regional boundaries.

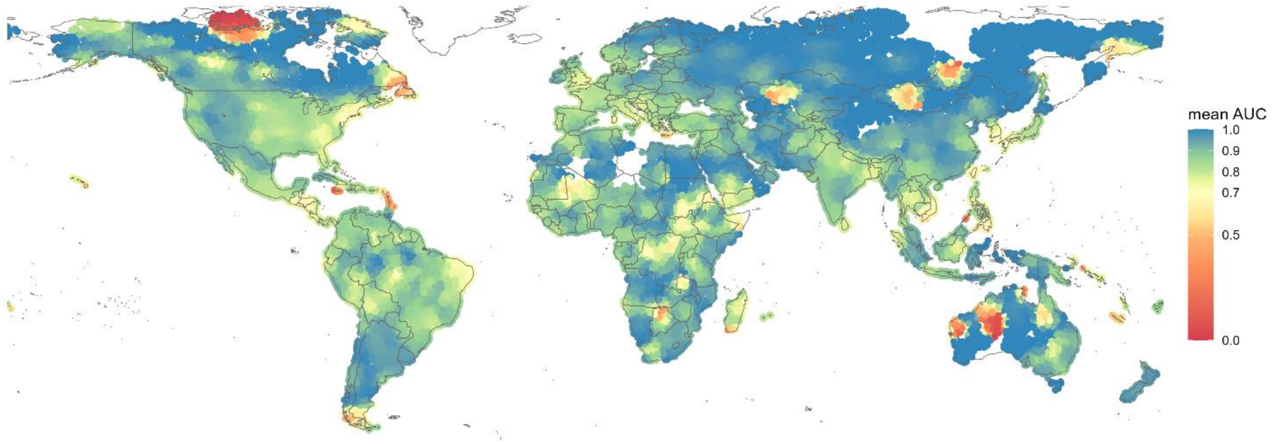

Supplementary Figure 7. Spatial map illustrating the model performance of the global surveillance capability model.

A spatial map of model performance, as measured by the Area Under the Curve (AUC), was created by calculating AUC within validation polygons (250 km radius around each presence or background (PB) point). ROC curves and AUC statistics were calculated for each polygon and summarised by centroid coordinates for visualisation. The map was created using public-domain Natural Earth data, accessed through the rnaturalearth package in R[32].

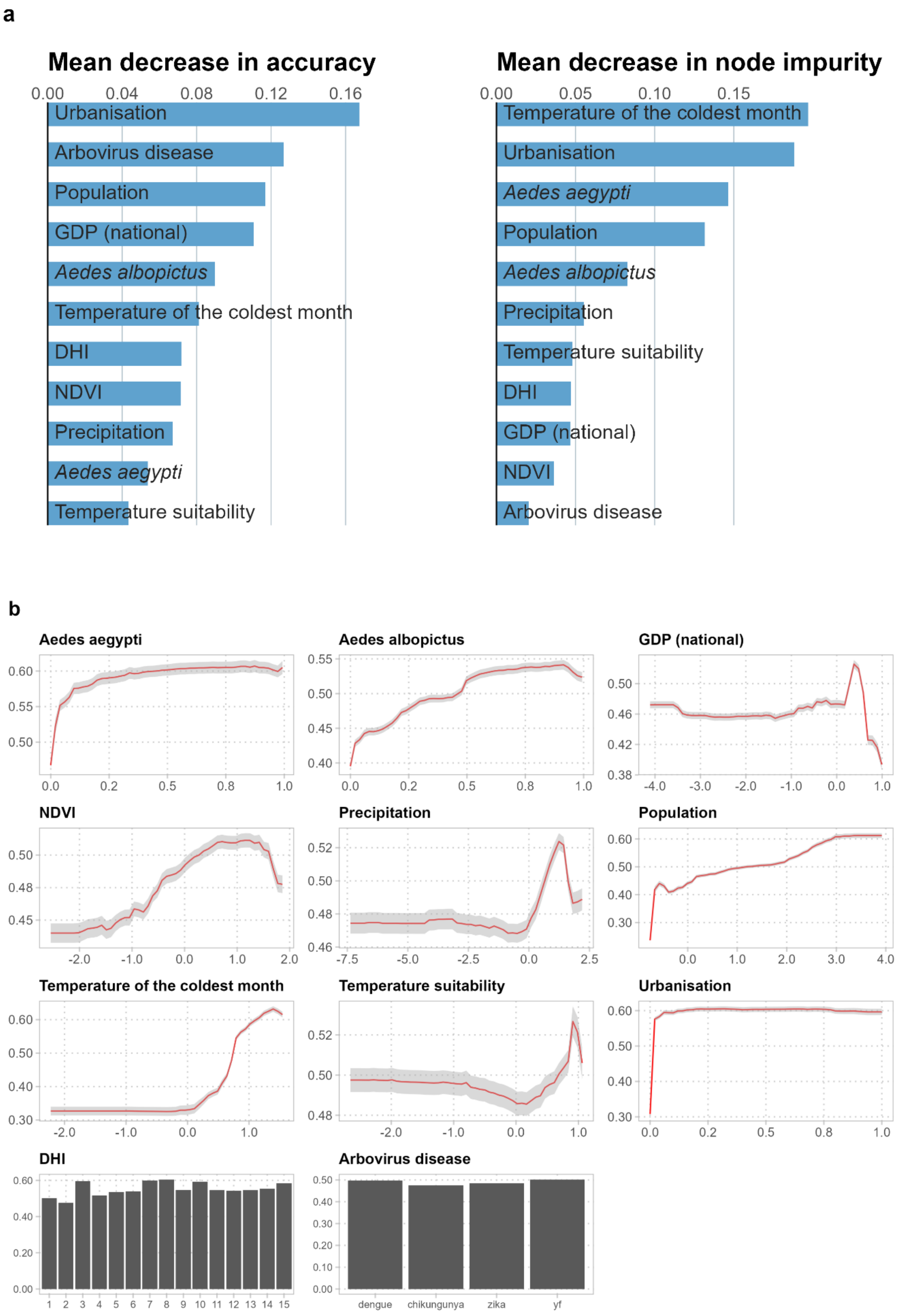

Supplementary Figure 8. Contribution of each covariate to the arbovirus model (dengue, chikungunya, and Zika).

Relative contribution of each covariate in explaining the global environmental suitability for dengue, chikungunya, and Zika (a); partial dependence plots showing the effects of each covariate on the overall response with 95% confidence intervals, where higher y-axis values indicate an increased probability of disease occurrence (b).

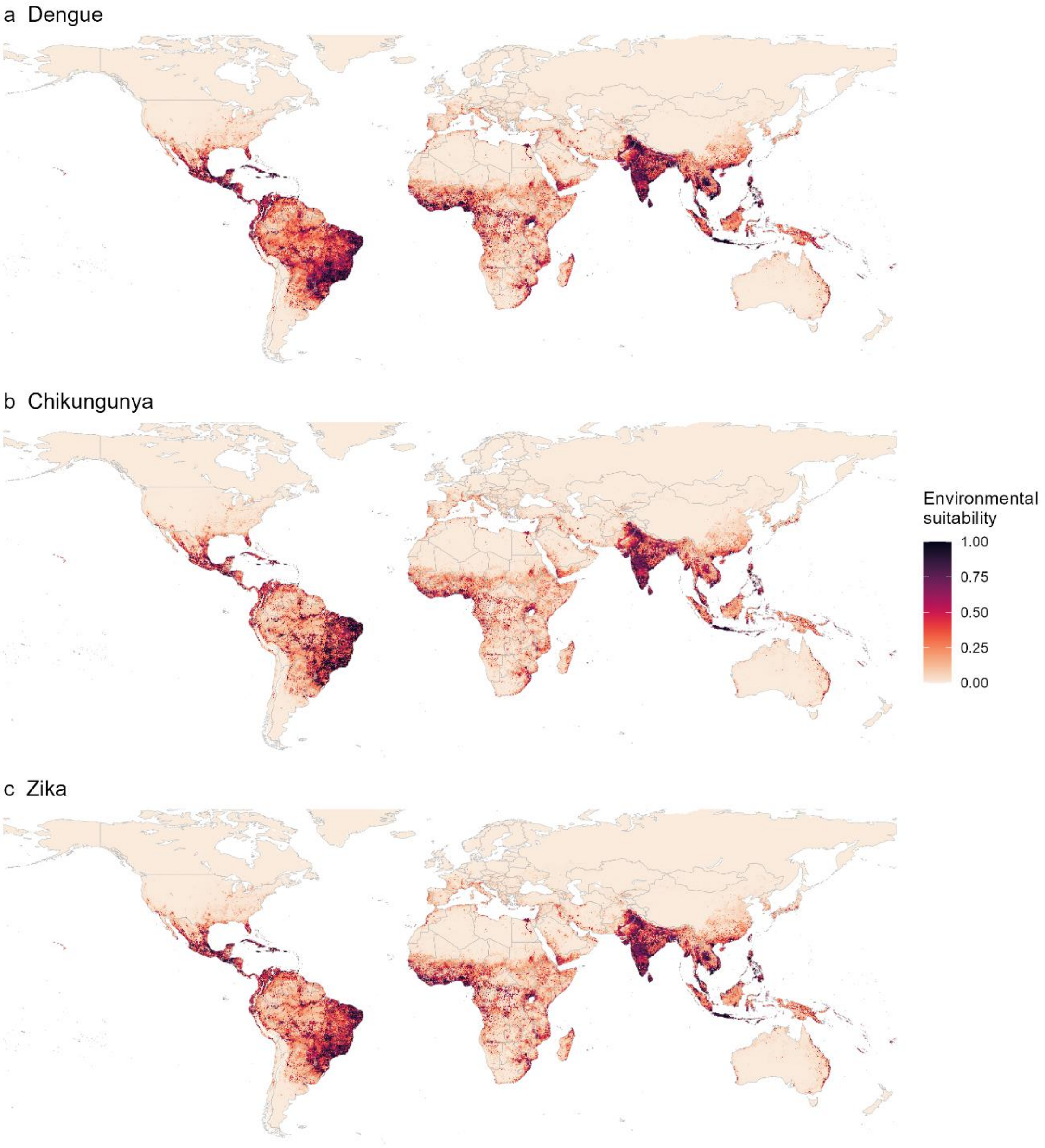

Supplementary Figure 9. Model predicted environmental suitability of each of dengue, chikungunya, and Zika.

Predicted environmental suitability for dengue (a), chikungunya (b), and Zika (c) from the arbovirus model. Areas outside the suitable temperature range for transmission are set to 0. These maps were combined into a unified map (Fig. 3a) by calculating the mean of each map. The maps were created using public-domain Natural Earth data, accessed through the rnaturalearth package in R[32].

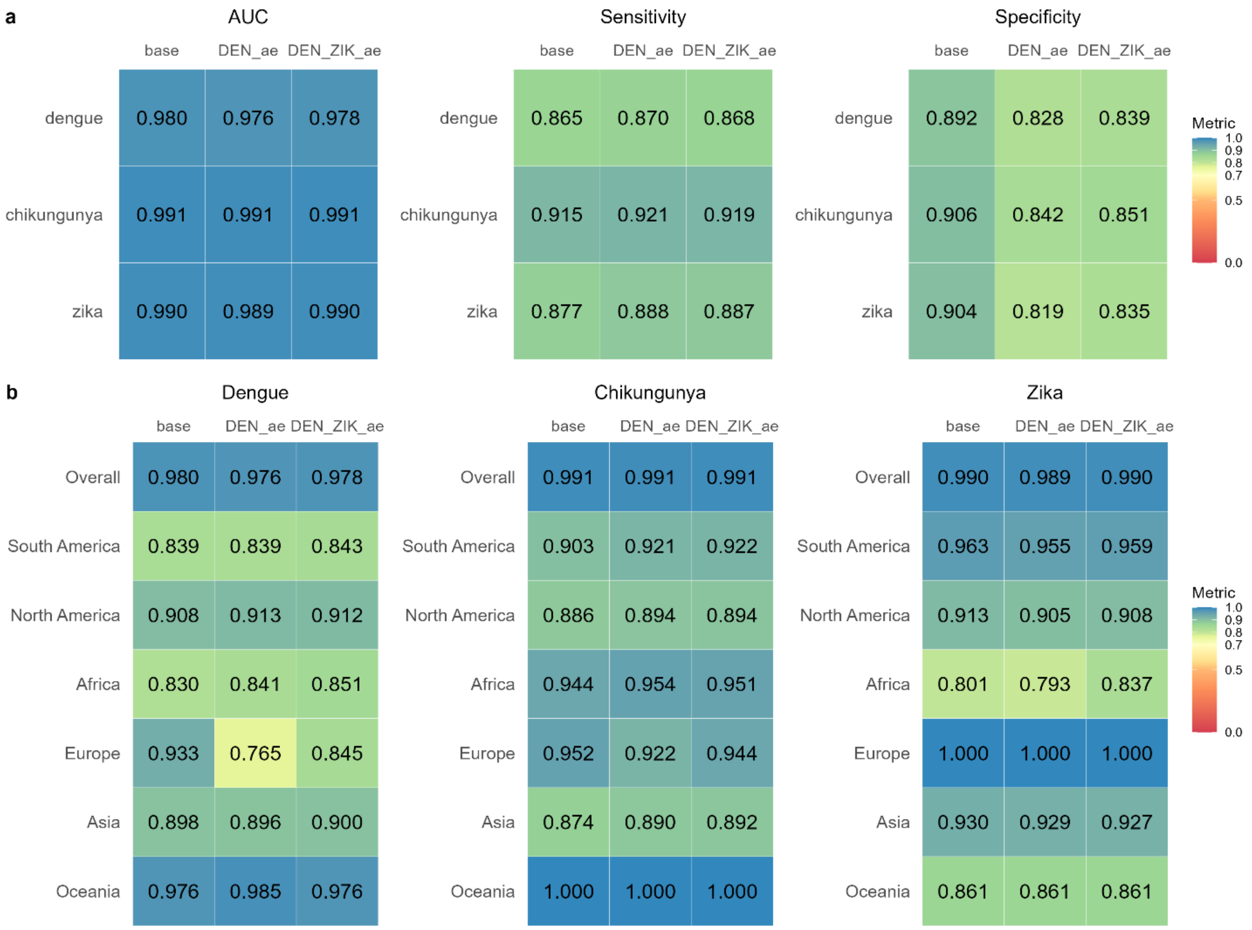

Supplementary Figure 10. A comparison of arbovirus model performance with and without disease-specific thermal suitability included as a covariate.

Heatmaps show the model performance metrics, including Area Under the Curve (AUC), sensitivity and specificity across the world, stratified by disease (a) and AUC only stratified by region and disease (b). We assess the performance of the base model (the model presented throughout the manuscript) against models incorporated alternative specifications between temperature and transmission risk[33,34]. Specifically, we compare the base model with two variants: one with the temperature suitability layer in the base model replaced by thermal suitability for dengue in *Aedes aegypti*[35] ("DEN_ae") and another including suitability layers for both dengue and Zika in *Ae. aegypti* ("DEN_ZIK_ae")[36]. Each model was assessed using a 50-fold block cross-validation approach, as detailed in the main text.

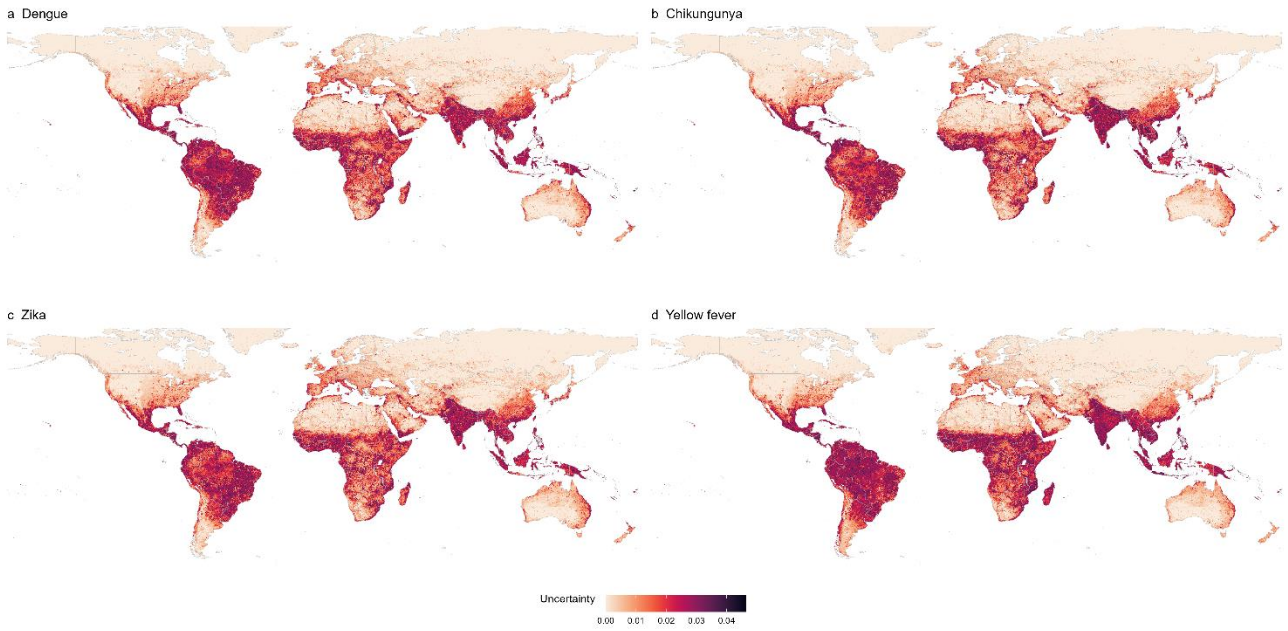

Supplementary Figure 11. The degree of uncertainty around arbovirus model predictions.

A total of 100 random forest sub-models were calibrated through spatial cross-validation to predict environmental suitability for dengue (a), chikungunya (b), Zika (c), and yellow fever (d) (0-1 scale). Model uncertainty was quantified by calculating the interquartile range (IQR) of the 100 model predictions at each location. The maps were created using public-domain Natural Earth data, accessed through the rnaturalearth package in R[32].

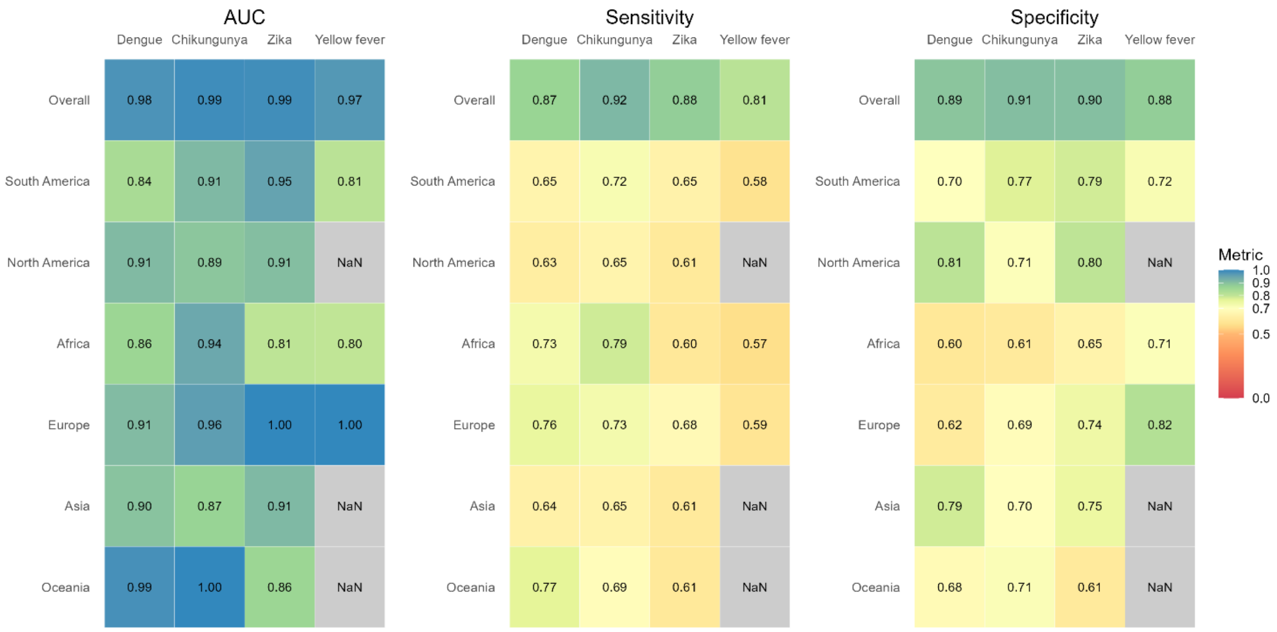

Supplementary Figure 12. Overall and regionally-stratified model performance metrics for the arbovirus and yellow fever models

Area Under the Curve (AUC), sensitivity, and specificity were calculated for each validation polygon (250 km radius around each presence or background point). Global and regionally stratified values for each metric were then derived by averaging these values within regional boundaries.

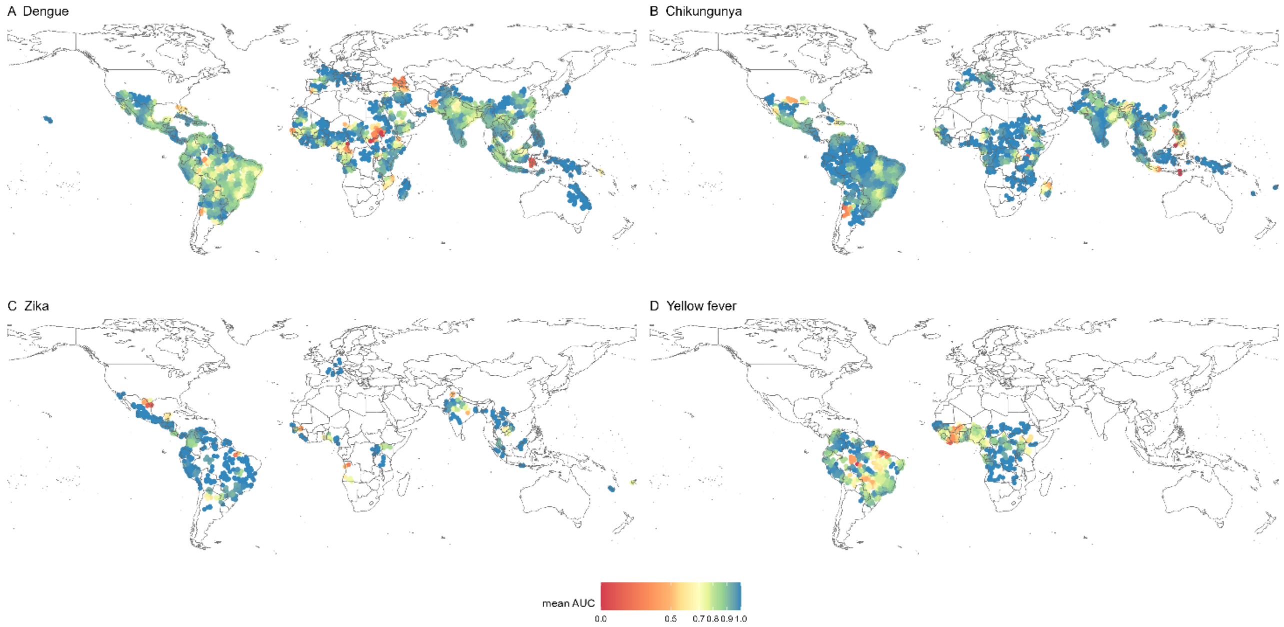

Supplementary Figure 13. Spatial map illustrating the model performance of the arbovirus model.

A spatial map of model performance, as measured by the Area Under the Curve (AUC), was created by calculating AUC within validation polygons (250 km radius around each presence or background (PB) point). ROC curves and AUC statistics were calculated for each polygon and summarised by centroid coordinates for visualisation. The maps were created using public-domain Natural Earth data, accessed through the rnaturalearth package in R[32].

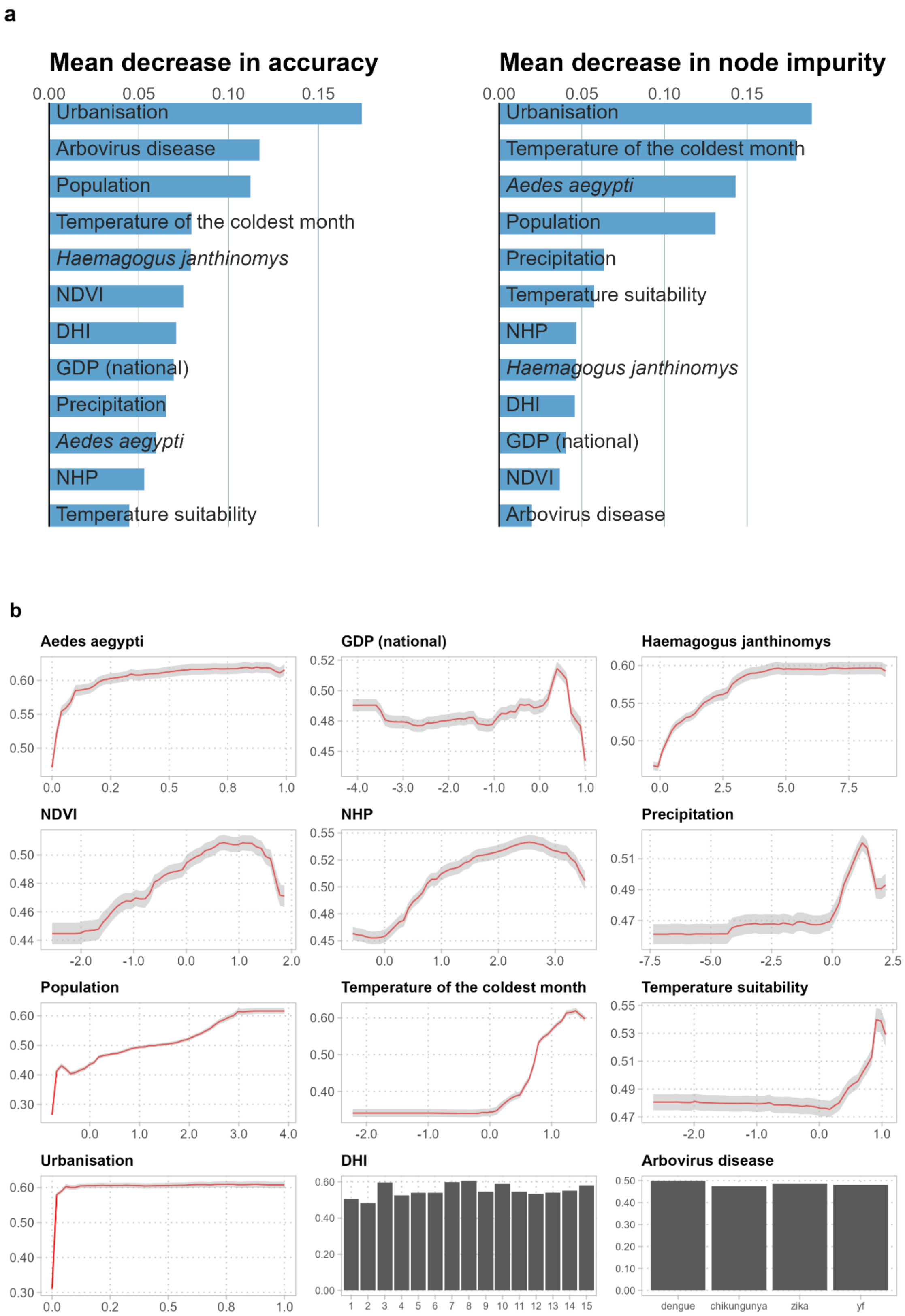

Supplementary Figure 14. Contribution of each covariate to the yellow fever model.

Relative contribution of each covariate in explaining the global environmental suitability for yellow fever (a); partial dependence plots showing the effects of each covariate on the overall response and their 95% confidence intervals (b).

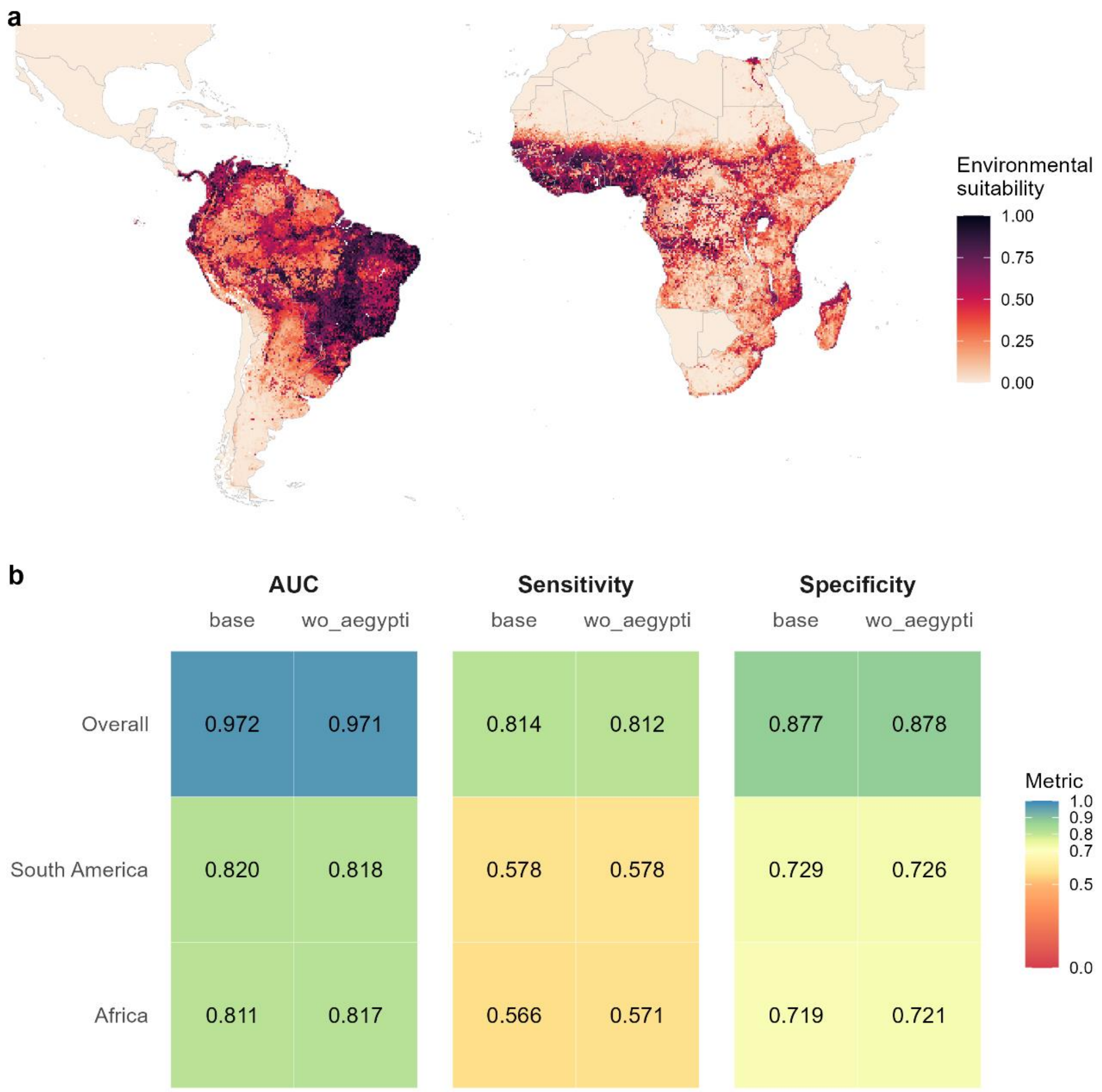

Supplementary Figure 15. A comparison of yellow fever model performance with and without *Aedes aegypti* included as a covariate.

Predicted environmental suitability of yellow fever after excluding the *Aedes aegypti* covariate from the yellow fever model (a). Heatmaps show the model performance metrics, including Area Under the Curve (AUC), sensitivity and specificity stratified by region (b). We compare the performance of the base model (as presented in the section "Environmental niche model") with an alternative version of model without *Ae. aegypti* covariate ("wo_aegypti"). Each model was assessed using a 50-fold block cross-validation approach, as detailed in the main text. The map was created using public-domain Natural Earth data, accessed through the rnaturalearth package in R[32].

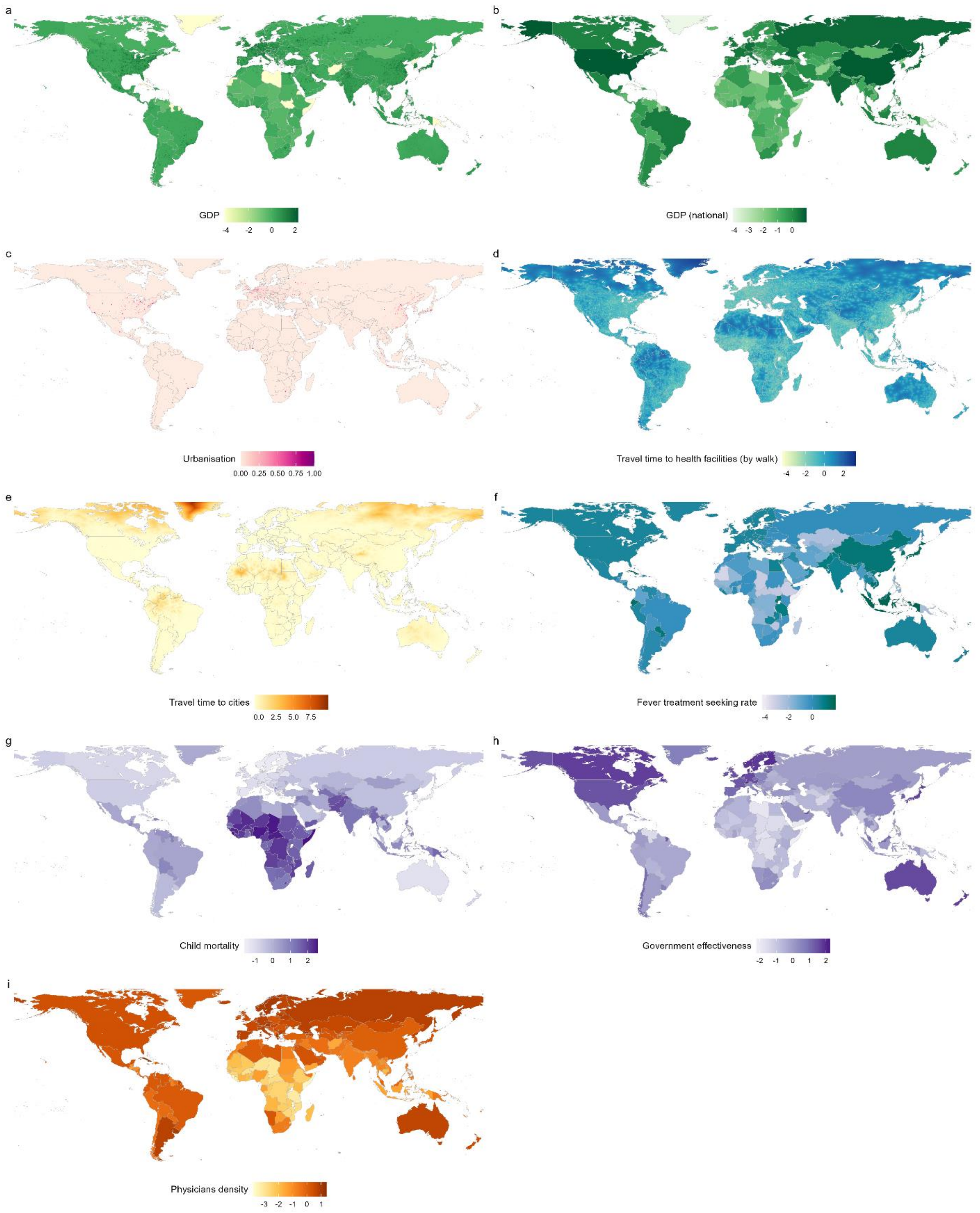

Supplementary Figure 16. Covariates included in the surveillance capability model.

(a-b) gross domestic product (GDP) (5 x 5 km resolution and aggregated national level)[2]; (c) the fraction of urban land[3]; (d) travel time to healthcare facilities by walk[4]; (e) travel time to cities (>50,000 people, any travel mode)[4]; (f) treatment-seeking for fever in children under five years old[5]; (g) child mortality under five years old[20,21]; (h) government effectiveness[6,722] and (i) physicians density[6,8]. Covariates were resampled to a consistent 0.05 degree grid with a common extent and land/sea mask with lakes and major water bodies removed. The log transformation was optionally applied based on the distributions of each covariate, and all covariates were scaled and centred to have a zero mean and variance of 1. The maps were created using public-domain Natural Earth data, accessed through the rnaturalearth package in R[32].

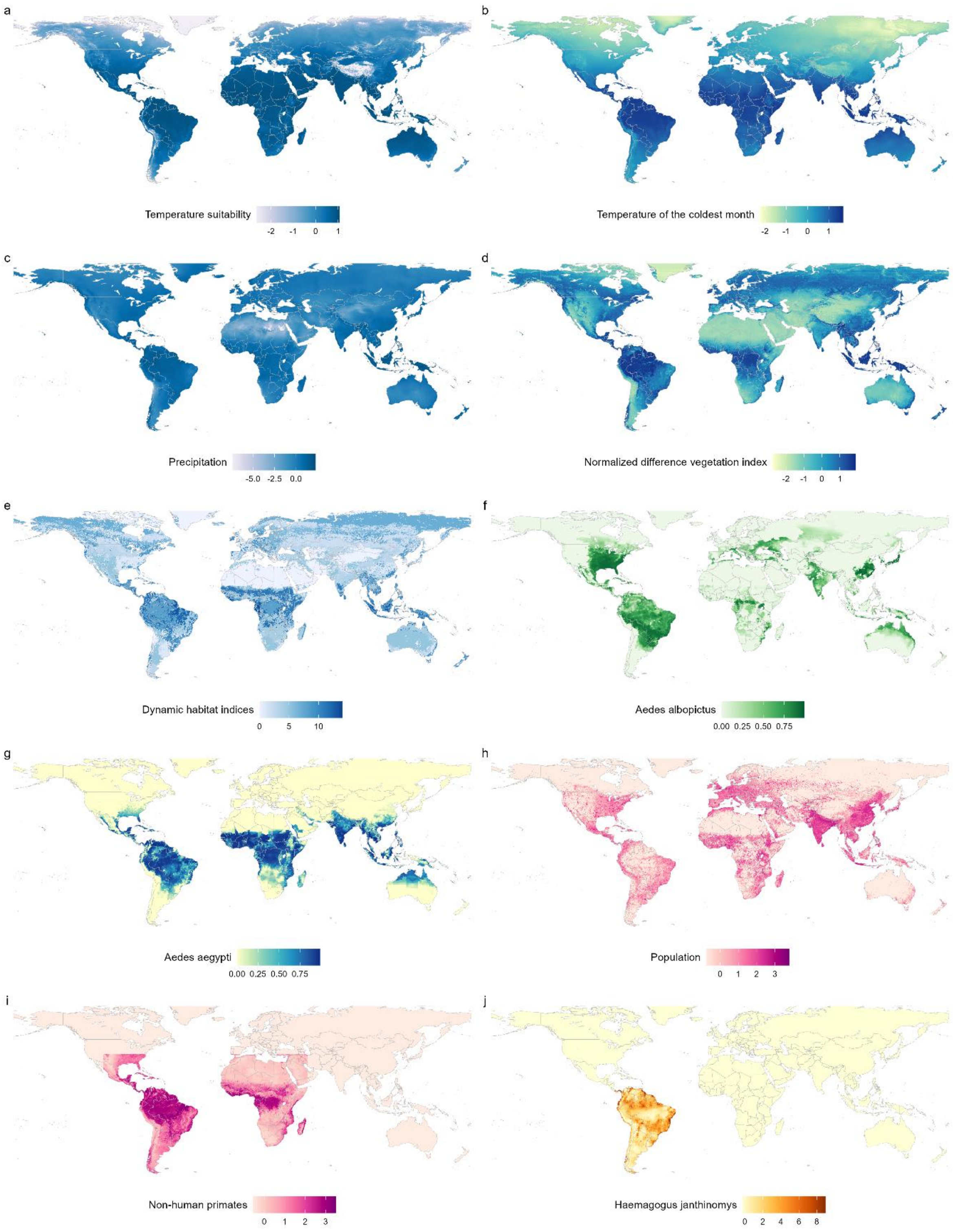

Supplementary Figure 17. Covariates included in the arbovirus and yellow fever models.

(a) temperature suitability for dengue virus transmission[24]; (b) mean temperature of the coldest month[259] (c) annual cumulative precipitation[9]; (d) Normalized Difference Vegetation Index (NDVI)[10]; (e) Dynamic Habitat Indices (DHI)[11]; (f) predicted suitability for *Ae. albopictus*[28]; (g) predicted suitability for *Ae. aegypti*[11]; (h) human population density[12]; (i) distribution of non-human primates (NHP)[13]; and (j) predicted suitability for *Haemagogus janthinomys* in South America[14]. Covariates were resampled to a consistent 0.05 degree grid with a common extent and land/sea mask with lakes and major water bodies removed. The log transformation was optionally applied based on the distributions of each covariate, and all covariates were scaled and centred to have a zero mean and variance of 1. The maps were created using public-domain Natural Earth data, accessed through the rnaturalearth package in R[32].

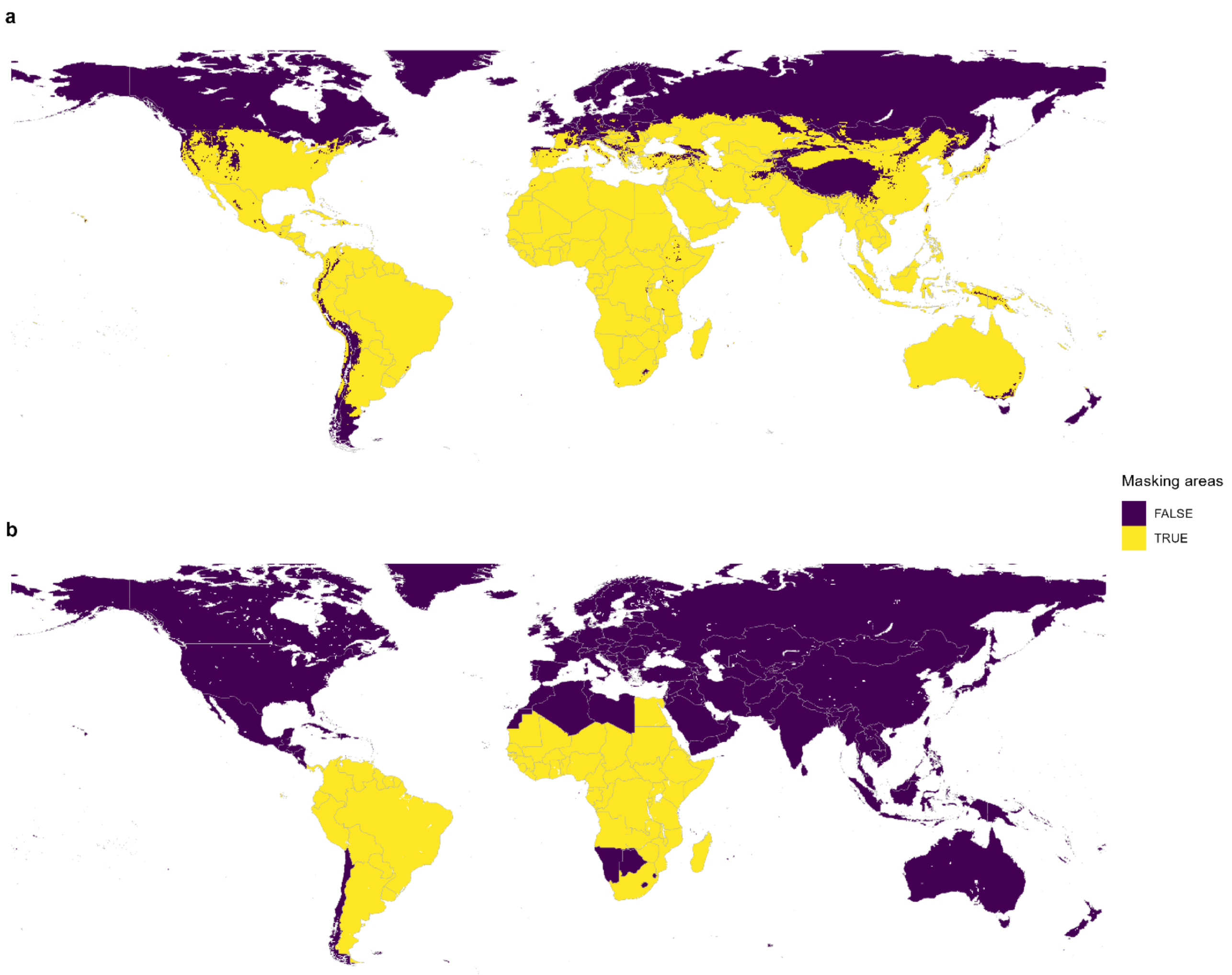

Supplementary Figure 18. Masking layers for arbovirus and yellow fever prediction.

Areas with unsuitable temperature ranges for dengue, chikungunya, and Zika (a), or outside the yellow fever (b) risk areas defined by the WHO yellow fever risk assessment working group, are set to 0 and indicated in purple. The maps were created using public-domain Natural Earth data, accessed through the rnaturalearth package in R[32].

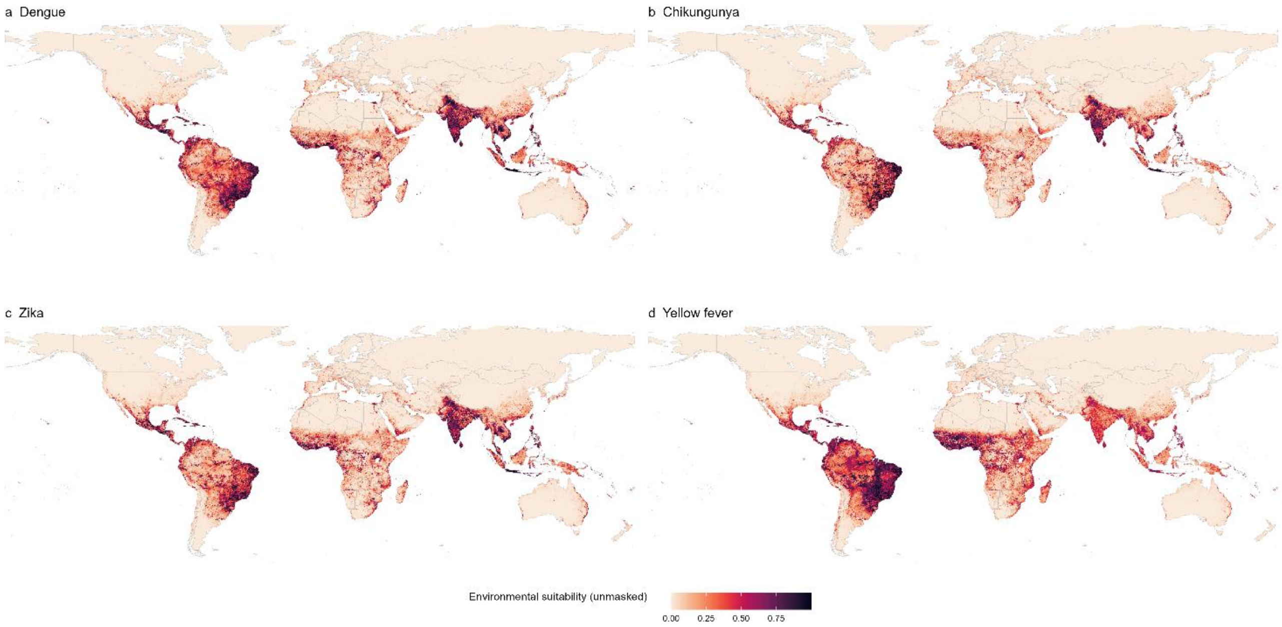

Supplementary Figure 19. Model predicted environmental suitability of arboviruses without masking.

Unmasked versions of environmental suitability maps for dengue (a), chikungunya (b), and Zika (c), and yellow fever (d). The maps were created using public-domain Natural Earth data, accessed through the rnaturalearth package in R[32].

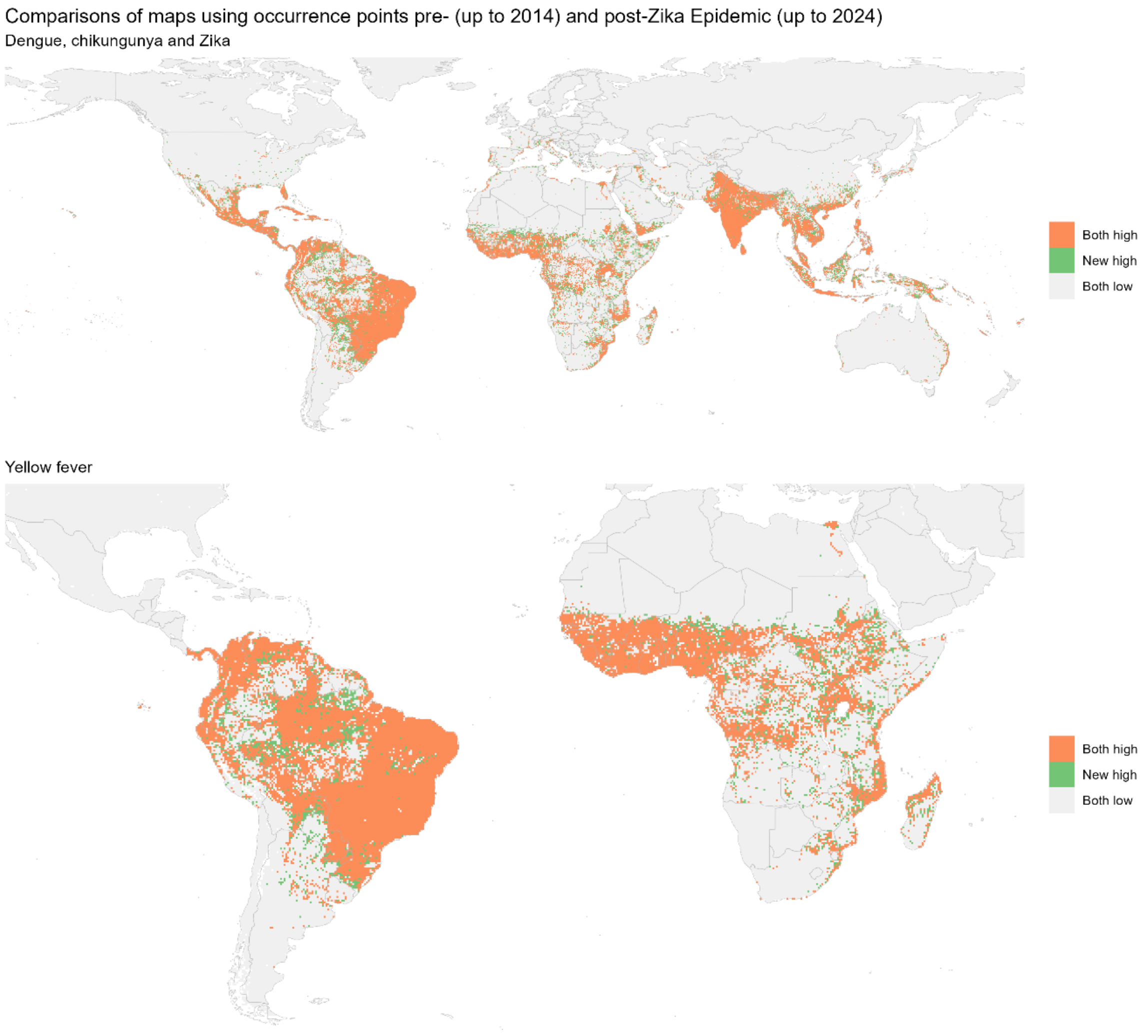

Supplementary Figure 20. Comparisons of maps using occurrence points pre- (up to 2014) and post-Zika epidemic (up to 2024)

While our primary focus is to model the environmental suitability (or "niche") of arboviral diseases rather than their temporal spread, we recognise the importance of testing whether the suitability predictions remain consistent over time.

To address this, we re-ran our models using occurrence data stratified into pre-Zika (up to 2014) and post-Zika (up to 2024) periods applying a 50-fold block cross-validation framework. Continuous suitability maps were converted into binary maps based on the threshold values calculated following the steps as detailed in the Methods section. This enabled us to compare areas classified as at-risk between the two time periods.

The comparison of the binary maps identified three categories: areas at-risk in both periods, areas at-risk in the post-Zika period only, and areas not at-risk in either period. While some new locations were classified as at-risk in the post-Zika map, the overall range of at-risk areas did not change significantly. These findings support our hypothesis that the environmental niche for these arboviral diseases has remained stable over time and that the observed geographic expansion reflects the diseases spreading into areas that were always environmentally suitable. The maps were created using public-domain Natural Earth data, accessed through the rnaturalearth package in R[32].

References

1. Shearer, F. M. *et al.* Existing and potential infection risk zones of yellow fever worldwide: a modelling analysis. *The Lancet Global Health* **6**, e270–e278 (2018).
2. Messina, J. P. *et al.* The current and future global distribution and population at risk of dengue. *Nat Microbiol* **4**, 1508–1515 (2019).
3. ECDC. Autochthonous vectorial transmission of dengue virus in mainland EU/EEA, 2010-present. https://www.ecdc.europa.eu/en/all-topics-z/dengue/surveillance-and-disease-data/autochthonous-transmission-dengue-virus-eueea (2023).
4. Disease Outbreak News. https://www.who.int/emergencies/disease-outbreak-news.
5. Frequent Import and Multiple Sources of Dengue Fever have Changed the Epidemic Situation of the Disease in Fujian Province, China. https://www.besjournal.com/en/article/doi/10.3967/bes2020.016.
6. Ren, J. *et al.* Epidemiology of Indigenous Dengue Cases in Zhejiang Province, Southeast China. *Front Public Health* **10**, 857911 (2022).
7. Yu, Y., Liu, Y., Ling, F., Sun, J. & Jiang, J. Epidemiological Characteristics and Economic Burden of Dengue in Zhejiang Province, China. *Viruses* **15**, 1731 (2023).
8. Sang, S., Yue, Y., Wang, Y. & Zhang, X. The epidemiology and evolutionary dynamics of massive dengue outbreak in China, 2019. *Front. Microbiol.* **14**, (2023).
9. Nsoesie, E. O. *et al.* Global distribution and environmental suitability for chikungunya virus, 1952 to 2015. *Eurosurveillance* **21**, 30234 (2016).
10. ECDC. Chikungunya worldwide overview. https://www.ecdc.europa.eu/en/chikungunya-monthly (2023).
11. ECDC. Autochthonous transmission of chikungunya virus in mainland EU/EEA, 2007–present. https://www.ecdc.europa.eu/en/infectious-disease-topics/z-disease-list/chikungunya-virus-disease/surveillance-threats-and (2019).
12. Wu, D. *et al.* Chikungunya Outbreak in Guangdong Province, China, 2010. *Emerg Infect Dis* **18**, 493–495 (2012).
13. Souza, W. M. de *et al.* Spatiotemporal dynamics and recurrence of chikungunya virus in Brazil: an epidemiological study. *The Lancet Microbe* **4**, e319–e329 (2023).
14. Messina, J. P. *et al.* Mapping global environmental suitability for Zika virus. *eLife* **5**, e15272 (2016).

15. Pan American Health Organization / World Health Organization (PAHO/WHO). Epidemiological Update - Yellow fever in the Region of the Americas - 25 April 2023. https://www.paho.org/en/documents/epidemiological-update-yellow-fever-region-americas-25-april-2023 (2023).
16. Chen, J. *et al.* Global 1 km × 1 km gridded revised real gross domestic product and electricity consumption during 1992–2019 based on calibrated nighttime light data. *Sci Data* **9**, 202 (2022).
17. Gao, J. & O'Neill, B. C. Mapping global urban land for the 21st century with data-driven simulations and Shared Socioeconomic Pathways. *Nat Commun* **11**, 2302 (2020).
18. Weiss, D. J. *et al.* Global maps of travel time to healthcare facilities. *Nature Medicine 2020 26:12* **26**, 1835–1838 (2020).
19. Nguyen, M. *et al.* Trends in treatment-seeking for fever in children under five years old in 151 countries from 1990 to 2020. *PLOS Global Public Health* **3**, e0002134 (2023).
20. United Nations Inter-agency Group for Child Mortality Estimation (UN IGME). *Levels and Trends in Child Mortality: Report 2022*. https://childmortality.org/wp-content/uploads/2023/01/UN-IGME-Child-Mortality-Report-2022.pdf.
21. INFORM Risk - Results and data.
22. Kaufmann, D. & Kraay, A. Worldwide Governance Indicators.
23. World Bank. Physicians (per 1,000 people).
24. Brady, O. J. *et al.* Global temperature constraints on Aedes aegypti and Ae. albopictus persistence and competence for dengue virus transmission. *Parasites & Vectors* **7**, 338 (2014).
25. Abatzoglou, J. T., Dobrowski, S. Z., Parks, S. A. & Hegewisch, K. C. TerraClimate, a high-resolution global dataset of monthly climate and climatic water balance from 1958–2015. *Sci Data* **5**, 170191 (2018).
26. Didan, K. MOD13C2 MODIS/Terra vegetation indices monthly L3 global 0.05 deg CMG V006. *NASA EOSDIS Land Processes DAAC* **10**, 2015 (2015).
27. Coops, N. C., Kearney, S. P., Bolton, D. K. & Radeloff, V. C. Remotely-sensed productivity clusters capture global biodiversity patterns. *Sci Rep* **8**, 16261 (2018).
28. Kraemer, M. U. G. *et al.* Past and future spread of the arbovirus vectors Aedes aegypti and Aedes albopictus. *Nat Microbiol* **4**, 854–863 (2019).
29. Sims, K. *et al.* LandScan Global 2022. Oak Ridge National Laboratory https://doi.org/10.48690/1529167 (2023).

30. Celone, M. *et al.* An ecological niche model to predict the geographic distribution of Haemagogus janthinomys, Dyar, 1921 a yellow fever and Mayaro virus vector, in South America. *PLOS Neglected Tropical Diseases* **16**, e0010564 (2022).

31. Hamlet, A. *et al.* POLICI: A web application for visualising and extracting yellow fever vaccination coverage in Africa. *Vaccine* **37**, 1384–1388 (2019).

32. South, A., Michael, S. & Massicotte, P. rnaturalearthdata: World Vector Map Data from Natural Earth Used in “rnaturalearth.” (2024).

33. Ryan, S. J., Carlson, C. J., Mordecai, E. A. & Johnson, L. R. Global expansion and redistribution of Aedes-borne virus transmission risk with climate change. *PLOS Neglected Tropical Diseases* **13**, e0007213 (2019).

34. Ryan, S. J. *et al.* Warming temperatures could expose more than 1.3 billion new people to Zika virus risk by 2050. *Glob Chang Biol* **27**, 84–93 (2021).

35. Ryan, S. Global current Aedes aegypti suitability for dengue transmission at 97.5% CI (5 arc minutes). Harvard Dataverse https://doi.org/10.7910/DVN/NSG5UH (2019).

36. Ryan, S. Global current suitability for Aedes aegypti transmitted Zika at 97.5% CI (5 arc minutes). Harvard Dataverse https://doi.org/10.7910/DVN/TK041G (2020).
