# Supplementary material for: The overlapping global distribution of dengue, chikungunya, Zika and yellow fever
Source: Nat Commun. 2025 Apr 10;16:3418. doi: 10.1038/s41467-025-58609-5 (PMC11986131; doi:10.1038/s41467-025-58609-5)
Supplement: Supplementary file 2 — Description of Additional Supplementary Files [file 41467_2025_58609_MOESM2_ESM.pdf]

Description of Additional Supplementary Files

File Name: Supplementary Data 1
Description: Average surveillance capability score and estimated number of people at risk, with 95% confidence interval bounds, for dengue, chikungunya, Zika, and yellow fever in each country.
