# Supplementary material for: The overlapping global distribution of dengue, chikungunya, Zika and yellow fever
Source: Nat Commun. 2025 Apr 10;16:3418. doi: 10.1038/s41467-025-58609-5 (PMC11986131; doi:10.1038/s41467-025-58609-5)
Supplement: Supplementary file 4 — Reporting Summary [file 41467_2025_58609_MOESM4_ESM.pdf]

nature portfolio

Corresponding author(s): Oliver J. Brady

Last updated by author(s): Mar 12, 2025

# Reporting Summary

Nature Portfolio wishes to improve the reproducibility of the work that we publish. This form provides structure for consistency and transparency in reporting. For further information on Nature Portfolio policies, see our Editorial Policies and the Editorial Policy Checklist.

## Statistics

For all statistical analyses, confirm that the following items are present in the figure legend, table legend, main text, or Methods section.

| n/a | Confirmed | |
|---|---|---|
| ☐ | ☒ | The exact sample size ($n$) for each experimental group/condition, given as a discrete number and unit of measurement |
| ☐ | ☒ | A statement on whether measurements were taken from distinct samples or whether the same sample was measured repeatedly |
| ☒ | ☐ | The statistical test(s) used AND whether they are one- or two-sided<br>*Only common tests should be described solely by name; describe more complex techniques in the Methods section.* |
| ☐ | ☒ | A description of all covariates tested |
| ☐ | ☒ | A description of any assumptions or corrections, such as tests of normality and adjustment for multiple comparisons |
| ☐ | ☒ | A full description of the statistical parameters including central tendency (e.g. means) or other basic estimates (e.g. regression coefficient) AND variation (e.g. standard deviation) or associated estimates of uncertainty (e.g. confidence intervals) |
| ☒ | ☐ | For null hypothesis testing, the test statistic (e.g. $F$, $t$, $r$) with confidence intervals, effect sizes, degrees of freedom and $P$ value noted<br>*Give P values as exact values whenever suitable.* |
| ☒ | ☐ | For Bayesian analysis, information on the choice of priors and Markov chain Monte Carlo settings |
| ☒ | ☐ | For hierarchical and complex designs, identification of the appropriate level for tests and full reporting of outcomes |
| ☒ | ☐ | Estimates of effect sizes (e.g. Cohen's $d$, Pearson's $r$), indicating how they were calculated |

*Our web collection on statistics for biologists contains articles on many of the points above.*

## Software and code

Policy information about availability of computer code

| | |
|---|---|
| Data collection | No primary data were collected as part of this study. All analyses were on secondary data with software and code used described in the Methods section. |
| Data analysis | Data analyses were carried out using R version 4.3.0 using the following packages: doParallel (v1.0.17), foreach (v1.5.2), pdp (v0.8.1), matrixStats (v1.3.0), Metrics (v0.1.4), cutpointr (v1.1.2), boot (v1.3-28.1), blockCV (v3.1-3), rsample (v1.2.0), randomForest (v4.7-1.1), terra (v1.7-29), rnaturalearth (v0.3.4), raster (v3.6-23), sp (v1.6-0), exactextractr (v0.9.1), sf (v1.0-14), tidyterra (v0.5.2), countrycode (v1.5.0), dplyr (v1.1.2), data.table (v1.14.8). All code and processed datasets used for the analyses are publicly available online in the study repository at https://github.com/ahyoung-lim/Arbo_riskmaps_public. |

For manuscripts utilizing custom algorithms or software that are central to the research but not yet described in published literature, software must be made available to editors and reviewers. We strongly encourage code deposition in a community repository (e.g. GitHub). See the Nature Portfolio guidelines for submitting code & software for further information.

## Data

Policy information about availability of data

All manuscripts must include a data availability statement. This statement should provide the following information, where applicable:

- Accession codes, unique identifiers, or web links for publicly available datasets
- A description of any restrictions on data availability
- For clinical datasets or third party data, please ensure that the statement adheres to our policy

Disease occurrence data is available from previous publications on dengue17, chikungunya18,38, Zika19, and yellow fever20. Additional data was extracted from publicly available sources, including WHO regional outbreak updates (https://www.who.int/emergencies/disease-outbreak-news); ECDC website (https://www.ecdc.europa.eu/en/); and ProMED mail reports (http://www.promedmail.org) and the HealthMap platform (www.healthmap.org). The maps with administrative boundaries were created using public-domain Natural Earth data, accessed via the rnaturalearth package in R86.

Climate and environmental covariates are freely available from previous publications (GDP 58, urbanization59, temperature suitability66, treatment-seeking for fever61, dynamic habitat indices69, and yellow fever vaccination coverage72). Surface travel time covariates are available from the Malaria Atlas Project (https://data.malariaatlas.org/maps). Child mortality, physicians density, and the government effectiveness estimates can be freely downloaded via European Commission Disaster Risk Management Knowledge Centre (https://drmkc.jrc.ec.europa.eu/inform-index/). High resolution population data can be freely obtained from LandScan program (https://landscan.ornl.gov/about). Global climate data can be downloaded from TerraClimate (https://www.climatologylab.org/terraclimate.html). Normalized Difference Vegetation Index data is freely available from NASA Earth Observation Data (https://www.earthdata.nasa.gov/) and can be downloaded using R MODIStp package (https://github.com/ropensci/MODIStsp). Predicted suitability for Ae. albopictus, Ae. aegypti, Haemagogus janthinomys, and non-human primates are not publicly available but can be obtained by contacting the authors of the cited papers20, 69, 71. A detailed description of data sources can be found in Supplementary Tables 1 and 4.

Processed versions of these datasets used in our analyses are available in two repositories: the study github repository (https://github.com/ahyoung-lim/Arbo_riskmaps_public) for past and current versions, and the Figshare repository (https://doi.org/10.6084/m9.figshare.26172934) for the version that has been peer-reviewed and described in this article.

## Research involving human participants, their data, or biological material

Policy information about studies with human participants or human data. See also policy information about sex, gender (identity/presentation), and sexual orientation and race, ethnicity and racism.

| | |
|---|---|
| Reporting on sex and gender | No analyses were performed on sex or gender because the data available to us did not disaggregate disease occurrences by these variables. |
| Reporting on race, ethnicity, or other socially relevant groupings | No analyses were performed on race, ethnicity, or other socially relevant groupings because the data available to us did not disaggregate disease occurrences by these variables |
| Population characteristics | N/A |
| Recruitment | N/A |
| Ethics oversight | N/A |

Note that full information on the approval of the study protocol must also be provided in the manuscript.

# Field-specific reporting

Please select the one below that is the best fit for your research. If you are not sure, read the appropriate sections before making your selection.

☐ Life sciences ☐ Behavioural & social sciences ☒ Ecological, evolutionary & environmental sciences

For a reference copy of the document with all sections, see nature.com/documents/nr-reporting-summary-flat.pdf

# Ecological, evolutionary & environmental sciences study design

All studies must disclose on these points even when the disclosure is negative.

| | |
|---|---|
| Study description | This is an observational mapping study that aims to characterise the relationship between several cliamtic and environmental variables and the probability of the arboviral disease occurrence. |
| Research sample | The primary data used in this study are occurrence records defined as unique geographic locations where one or more cases of a particular disease have been reported at any point in time [21]. Occurrence data were restricted to locally-acquired infections only, as different factors may influence the risk of importation and establishment in transmission-free areas, potentially requiring different modeling approaches. The dataset represents regions where the diseases have ever been circulating, and the data were sourced |

| | |
|---|---|
| | from previous publications on dengue17, chikungunya18,38, Zika19, and yellow fever20 as well as ProMed mail, ECDC, and WHO Outbreak News. |
| Sampling strategy | Because no hypotheses were statistically compared, no sample size calculation was required. Instead we aimed to use the maximum amount of data available over a period where occurrence data is available to maximize our chances of characterizing the important relationships in our model. As data on arboviral disease absence is difficult to collect, standardize and validate, we used a "presence-background" modeling approach [57] where absence or background points are randomly generated across all land surfaces. Here the number of background points selected was proportionate to the number of occurrence data for each disease separately in order to maintain a 1:1 ratio between presence and background points (the approach is often referred to as "down-sampling [56, 73]"). |
| Data collection | Occurrence data were downloaded from previous publications[17-20,38] between 10 -16 April, 2023. Additional data were collected from ProMed Mail between 6 February and 26 April 2023 using an Excel spreadsheet. Additional data searches were conducted for ECDC, WHO regional outbreak updates on 10 April 2024. There was no restrictions on the spatial scale from which the data were collected. |
| Timing and spatial scale | The dataset includes historical disease occurrence data, resulting in a total of 58,361 records of dengue, chikungunya, Zika, and yellow fever occurrences between 1927 and March 2024. These records encompass observations from across the entire world. |
| Data exclusions | Travel-related and imported cases were excluded from occurrence data. No other exclusions were applied. |
| Reproducibility | Results were verified independently by two different authors (AL and OB). Internal cross valdiation experiments (both temporal and spatial) also tested generalizability of the findings. |
| Randomization | Not relevant to this study as it is an observational study with no intervention or experimental groups. |
| Blinding | Blinding was not possible in this study as it is a secondary analysis of observational data. |

Did the study involve field work? ☐ Yes ☒ No

# Reporting for specific materials, systems and methods

We require information from authors about some types of materials, experimental systems and methods used in many studies. Here, indicate whether each material, system or method listed is relevant to your study. If you are not sure if a list item applies to your research, read the appropriate section before selecting a response.

## Materials & experimental systems

| n/a | Involved in the study | |
|---|---|---|
| ☒ | ☐ | Antibodies |
| ☒ | ☐ | Eukaryotic cell lines |
| ☒ | ☐ | Palaeontology and archaeology |
| ☒ | ☐ | Animals and other organisms |
| ☒ | ☐ | Clinical data |
| ☒ | ☐ | Dual use research of concern |
| ☒ | ☐ | Plants |

## Methods

| n/a | Involved in the study | |
|---|---|---|
| ☒ | ☐ | ChIP-seq |
| ☒ | ☐ | Flow cytometry |
| ☒ | ☐ | MRI-based neuroimaging |

## Plants

| | |
|---|---|
| Seed stocks | N/A |
| Novel plant genotypes | N/A |
| Authentication | N/A |
